# F83. INVESTIGATING THE RELATIONSHIP BETWEEN NEGATIVE SYMPTOM PROFILE AND COGNITIVE FUNCTION IN SCHIZOPHRENIA

**DOI:** 10.1093/schbul/sby017.614

**Published:** 2018-04-01

**Authors:** Caitlin Yolland, Wei Lin Toh, Eric Tan, Caroline Gurvich, Erica Neill, Susan Rossell

**Affiliations:** 1 Swinburne University of Technology; 2 Monash Alfred Psychiatry Research Centre; 3 Brain and Psychological Sciences Research Centre, Swinburne University, Monash Alfred Psychiatry Research Centre, The Alfred Hospital and Monash University; 5 University of Melbourne

## Abstract

**Background:**

Negative symptoms are core to schizophrenia. Understanding the complex way specific symptom profiles may affect cognition independent of a diagnosis of schizophrenia per se will allow for an improved understanding of the disorder, and specific subtypes as well as potential treatment targets therein.

**Methods:**

The neurocognitive profiles of 132 patients with schizophrenia/schizoaffective disorder and 189 healthy controls were examined using the MATRICS Consensus Cognitive Battery. Patients were grouped as either having a negative symptom profile or no negative symptoms using the PANSS. Healthy controls were grouped as high or low schizotypy on the negative symptom analogue subscale from the O-LIFE.

**Results:**

There was a significant effect of negative symptom profile on the processing speed domain, the participants with negative symptoms performed significantly worse than those with no negative symptoms, after controlling for premorbid IQ, F(1,129)=4.30, p<0.05. The same relationship with speed of processing was found when investigating high vs low schizotypal aspects of negative symptoms in an equivalent analysis of healthy controls, with those scoring highly on negative symptoms performing significantly worse, after premorbid IQ was controlled for, F(1,186)=6.24, p<0.05.

**Discussion:**

The processing speed domain seems significantly impacted by negative symptom profile in both schizophrenia patients and healthy controls. The speed of processing deficits does not seem to be presenting a bottom up influence on higher order cognitive tasks, as no group differences were observed on reasoning and problem solving tasks. In conclusion, these findings indicate that the negative symptom cluster contributes to this specific cognitive impairment independently of the disorder.

